# Fracture of the Humerus, without the Application of Violence

**Published:** 1846-07

**Authors:** J. Berncastle

**Affiliations:** M. D., M. R. C. S. Eng., Croydon


*Fracture of the Humerus, without the application of Violence.* By J. Berncastle, M. D., M. R. C. S. Eng., Croydon.—The following instance of almost spontaneous fracture of the humerus occurred to a man named Williams, aged thirty, an excavator on the Epsom line of railway. He was in the act of lifting from the ground with his left hand a piece of wood about a foot in length, and not weighing more than ten pounds, intending to place it on his right shoulder, when he suddenly heard something snap and break. He applied immediately for advice; and on examination, the left humerus was found to have sustained a complete transverse fracture at about the middle of the shaft of the bone. A fracture having taken place from such an unusual cause, led me to inquire minutely into the history of the man, and I found, that six years ago he had gonorrhœa, for which he salivated himself. Three years back, he was laid up with rheumatism at Guy's Hospital, in Billet ward, under Mr. Cock, and came out cured. A year ago, he had rheumatism of the right arm; and six weeks before the late accident, he had had a severe attack in the left arm, now broken, which was treated with leeches, blisters, &c. All this does not appear in any way to have been likely to produce a predisposition to fracture; and even supposing that there might have been a diseased state of bone masked by the rheumatic symptoms, that suppposition is quite done away with by the fact of the bone having perfectly united, without a single troublesome symptom, in the space of one month, the callus being distinctly felt around the part, and the man able to use his arm, showing that the bone is in a perfectly normal state. He expresses himself quite pleased with the accident, because he says he has been easy ever since from the rheumatic pains—rather a novel "modus curandi!" Being a strong, athletic man, anything like a tendency to mollities ossium is out of the question; and the small size of the piece of wood he was in the act of lifting when the fracture occurred, rendering the theory of violent muscular contraction also inapplicable, I am at a loss to account for the occurrence at all in a satisfactory manner, and I consider it a most rare and singular case of fracture.—*London Lancet.*

